# Ecology and distribution of large branchiopods (Crustacea, Branchiopoda, Anostraca, Notostraca, Laevicaudata, Spinicaudata) of the Eastern Cape Karoo, South Africa

**DOI:** 10.3897/zookeys.618.9212

**Published:** 2016-09-19

**Authors:** Annah Mabidi, Matthew S. Bird, Renzo Perissinotto, D. Christopher Rogers

**Affiliations:** 1 DST/NRF Research Chair: Shallow Water Ecosystems, Nelson Mandela Metropolitan University, P.O. Box 77000, Port Elizabeth 6031, South Africa; 2 Africa Earth Observatory Network, Nelson Mandela Metropolitan University, P.O. Box 77000, Port Elizabeth 6031, South Africa; 3 Kansas Biological Survey/Biodiversity Institute, Kansas University, Higuchi Hall, 2101 Constant Avenue, Lawrence, KS 66047-3759, USA

**Keywords:** Depression wetlands, environmental monitoring, hydraulic fracturing, invertebrate biogeography, wetland invertebrates

## Abstract

A survey of the large branchiopod fauna of the Eastern Cape Karoo region of South Africa was undertaken to provide baseline biodiversity information in light of impending shale gas development activities in the region. Twenty-two waterbodies, including nine dams and thirteen natural depression wetlands, were sampled during November 2014 and April 2015. A total of 13 species belonging to four orders were collected, comprising five anostracans, one notostracan, six spinicaudatans and one laevicaudatan. *Cyzicus australis* was most common, occurring in 46% of the waterbodies. Species co-occurred in 87% of the waterbodies, with a maximum number of six species recorded from the same waterbody. Our new distribution records for *Lynceus truncatus*, *Streptocephalus spinicaudatus* and *Streptocephalus indistinctus* represent substantial expansions of the previously known ranges for these species. Tarkastad is now the westernmost record for *Streptocephalus spinicaudatus*, while Jansenville now constitutes the southernmost record for *Streptocephalus indistinctus*. Large branchiopod distribution data from previous Eastern Cape records were combined with our current data, demonstrating that a total of 23 large branchiopod species have been recorded from the region to date. As the Karoo is one of the few major shale basins in the world where the natural baseline is still largely intact, this survey forms a basis for future reference and surface water quality monitoring during the process of shale gas exploration/extraction.

## Introduction

Large branchiopod crustaceans belonging to the orders Anostraca (fairy shrimps), Notostraca (tadpole shrimps), Laevicaudata (smooth clam shrimps) and Diplostraca (suborder Spinicaudata, spiny clam shrimps) are obligatory residents of temporary waterbodies throughout the world (Day et al. 1999, Brendonck et al. 2008, Rogers 2009). Large branchiopods are adapted to these systems and survive drought phases as dormant eggs which can remain in the sediments of a dry wetland for many years (Wiggins et al. 1980, Rogers 2015a). The dormant eggs hatch during favourable environmental conditions and only a fraction of the resting stages hatch per each inundation (Brock et al. 2005, Rogers 2015a, b). This is a bet–hedging strategy aimed at ensuring long-term survival of populations (Brendonck and De Meester 2003, Schwentner and Richter 2015, Rogers 2015a, b). Most large branchiopods are filter feeders, which indiscriminately filter particles from water (Brendonck et al. 2008). However, the notostracans and a few anostracans are omnivorous and predatory as adults (Rogers 2009).

Large branchiopods are primarily restricted to rain-fed (as opposed to groundwater-fed) temporary aquatic habitats, such as ephemeral rock pools, natural depressional wetlands, roadside ditches, farm dams and pools in riverbeds that dry completely in the warm months (Brendonck et al. 2008, Rogers 2009). Ecologically, these temporary aquatic habitats are among the most extreme aquatic environments, with highly variable physico-chemical conditions that vary both during inundations and between inundation events (Meintjes et al. 1994, Williams 2006, Boven et al. 2008, Nhiwatiwa et al. 2011, Rogers 2014b). They are also among the most seriously threatened habitats globally (Semlitsch and Bodie 1998), among other factors due to their relatively small volume and shallow depth, which make them easy targets for infilling, drainage and rapid pollution (Hamer and Brendonck 1997, Williams 2006, Darwall and Brooks 2011, Collen et al. 2014).

Factors influencing large branchiopod assemblages have been studied extensively (Hamer and Appleton 1991a, b, Thiéry and Puente 2002, Timms and Sanders 2002, Boven et al. 2008, Waterkeyn et al. 2008, Rogers 2009, Padhye and Dahanukar 2015). Annual average rainfall, rainfall season and effective temperature are climatic factors that appear to influence anostracan distribution (Hamer and Brendonck 1997), while local abiotic factors such as waterbody size, number of niches, habitat duration and life history traits influence large branchiopod species richness (Thiéry 1991, Hamer and Appleton 1991b, King et al. 1996, Waterkeyn et al. 2009). Relationships between geochemical substrate properties and the distribution of anostracan species have also been reported (Rogers 2014).

Sixty-six large branchiopod species have been documented within the southern African region to date (Day et al. 1999, Rogers 2013). However, large branchiopod crustaceans are still among the least known of all macroinvertebrates in temporary inland waters of the region and richness is expected to be substantially higher, given that vast areas of the southern African subcontinent remain unstudied (Brendonck et al. 2008). South Africa is no exception, as there is currently limited information on large branchiopod species distribution and their relationships with habitat factors (Hamer and Brendonck 1997, Henri et al. 2014). Information about the distribution and conservation status of large branchiopod species is available for some areas of South Africa. These include the KwaZulu-Natal lowlands (Rayner and Bowland 1985a, Hamer and Appleton 1991a), the mountainous Drakensberg region of KwaZulu-Natal (Hamer and Martens 1998), the Northen Cape (Hamer and Rayner 1996), Western Cape (De Roeck et al. 2007b, 2010), North West and Free State provinces (Henri et al. 2014), as well as the Mpumalanga Highveld region (Riato et al. 2014). Virtually nothing, however, is known about the large branchiopods living in the semi-arid Karoo basin. The known large branchiopod distribution records in this region are few and were mainly obtained from a single sampling expedition in 1996 (Day et al. 1999). This lack of information is of concern, particularly given that the region has recently been earmarked for shale gas exploration through hydraulic fracturing methods (Econometrix 2012, Geel et al. 2015, Tucker and van Tonder 2015, Murray et al. 2015). The hydraulic fracturing process uses large amounts of water and in turn produces large amounts of briny waste water, which when mismanaged may pollute both surface and groundwater systems (McBroom et al. 2012, Mauter et al. 2014). This may compound problems associated with water scarcity in this naturally arid region, which is already experiencing drier conditions as a result of climate change (Hewitson and Crane 1996, Cubasch et al. 2013). Large branchiopods, as obligatory residents of temporary waterbodies, are expected to be among the most threatened by activities associated with shale gas exploration and development through hydraulic fracturing. The inhabitants may be affected by contamination from leakage and spillage of insufficiently treated wastewater and chemicals, sedimentation due to development of additional roads, and landscape fragmentation (Warner et al. 2013, Mauter et al. 2014).

Here, we present large branchiopod diversity and distribution patterns prior to shale gas development. We present data on patterns of species assemblage composition and richness and assess these patterns in the context of environmental parameters for regional branchiopod populations. The survey is the first of its kind for the Karoo region and represents an important step towards understanding large branchiopod communities in this largely unexplored region. This information can be useful in planning and decision-making for development and to monitor changes in the temporary aquatic biota of the region in relation to future impacts.

## Materials and methods

### Study area

**Figure 1.**
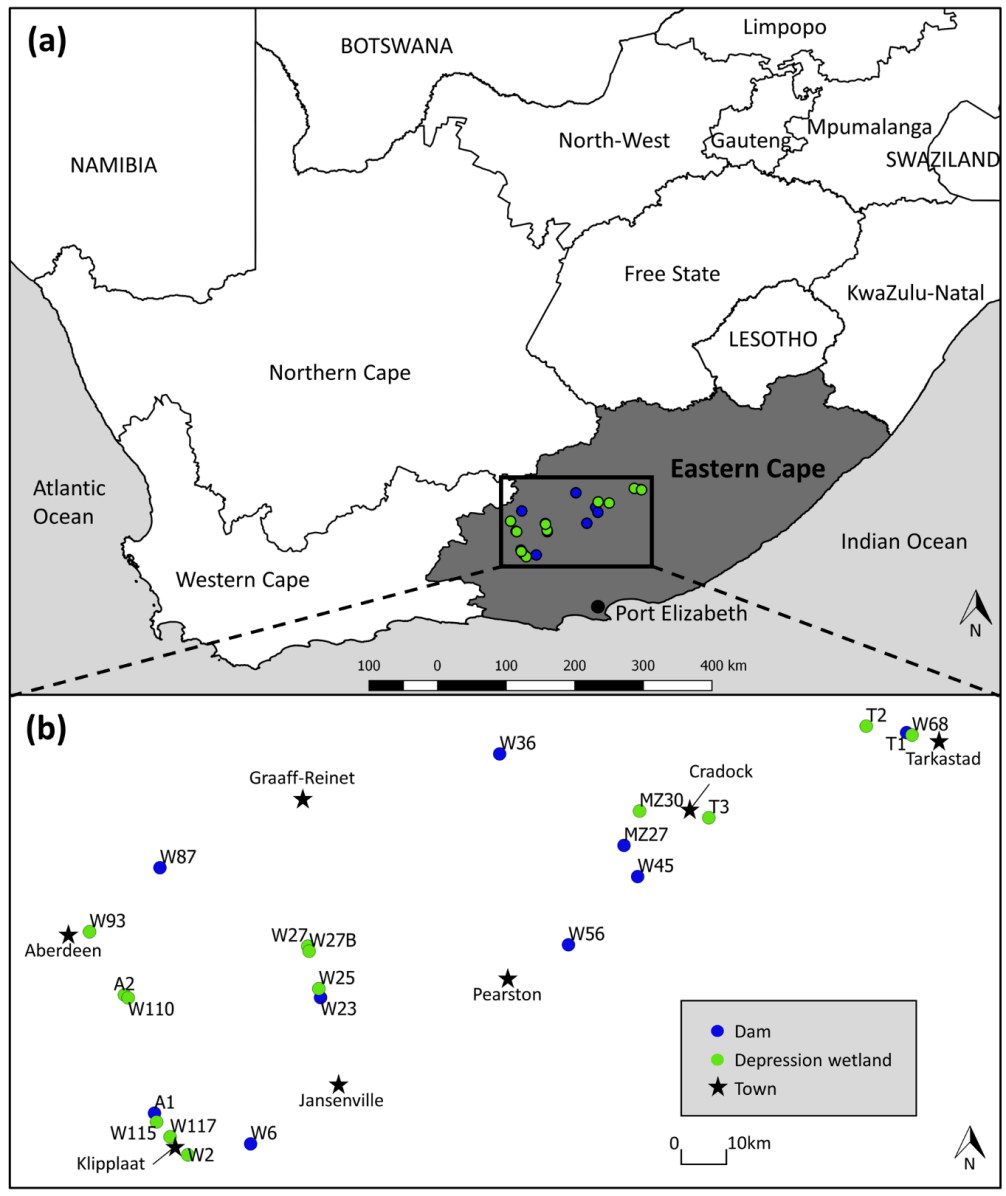
Location of the sites sampled for large branchiopods in the Eastern Cape Karoo region of South Africa (**a**), including a zoomed-in perspective of the 22 sites within the focal study area (**b**).

The study area occurs within the Eastern Cape Province of South Africa (Figure 1). Air temperatures in the region are notoriously variable both diurnally and seasonally. Temperature extremes range from -5 °C in winter (mean July daily minimum < 0 °C) to 43 °C in summer (mean January daily maximum > 30 °C) (Schulze 1997, Mucina et al. 2006). Regional rainfall is highly unpredictable in both space and time. Sporadic rainfall events occur throughout the year, displaying elements of a perennial precipitation regime. However, long term records show that the bulk of the rainfall generally occurs in summer, peaking between December and March (Schulze et al. 1997). Mean annual precipitation ranges from 70 mm in the west to around 400 mm in the east, with a coefficient of variation of annual precipitation of 30–60% (Schulze 1997, Desmet and Cowling 1999). The hot and dry climatic conditions limit the occurrence of perennial aquatic habitats, whilst favouring the presence of temporary waterbodies.

The waterbodies of the region can be divided into three major types. First are depressional systems, which manifest as surface water on a temporary basis predominantly as isolated pools fed by direct precipitation, although some depressions may be connected to a larger drainage network (Ollis et al. 2015). Second are rivers (longitudinal features), which are generally small and temporarily inundated. Very few river systems in the region are genuinely permanent (e.g. Great Fish River). Lastly, dams (reservoirs) provide an exception to the natural surface water regime of the region in that they artificially increase the number of permanent water features in the landscape. These reservoirs are typically utilized for livestock.

Low intensity rangeland agriculture for livestock grazing is the main land-use activity in the region, although sparse amounts of irrigation agriculture and mining do occur (Mucina and Rutherford 2006). The regional geology is characterised by shallow, weakly developed lime-rich soils underlain by Ecca and Beaufort shales of the Karoo Supergroup (mostly glacial, shale and sandstone deposits, van Tonder et al. 2013). Our study area lies in the Nama Karoo biome, between 384 and 1450 m elevation and is dominated by low-shrub vegetation (< 1m tall) intermixed with grasses, succulents, geophytes and annual forbs (Dean and Milton 1999, Meadows and Watkeys 1999, Mucina et al. 2006).

### Sampling sites and methods

Twenty-two lentic habitats (nine dams and thirteen depressional wetlands) were sampled for large branchiopods during November 2014 (austral spring) and April 2015 (austral autumn, see Suppl. material 1: Appendix 1 for full locality information for each habitat). The geographic region covered includes the Eastern Cape Karoo area earmarked for shale gas exploration, specifically from the towns of Aberdeen and Jansenville in the west to Tarkastad and Cradock in the east (Figure 1). Site W117 (depression wetland) was only sampled in November 2014, as it was dry during the April 2015 survey. The sites were divided into three size categories according to surface area, as small (< 499 m^2^), medium (500–1000 m^2^) and large (> 1000 m^2^). Large branchiopods were sampled semi-quantitatively with a D-frame sweep net (1 mm mesh size, 250 mm mouth diameter) by means of a timed collection effort standardised according to the three size categories. Small wetlands were swept for three minutes, medium-sized wetlands for six minutes and large wetlands for twelve minutes. The samples were preserved in a 10% formalin solution. Material was identified to lowest justifiable taxonomic level using keys by Day et al. (1999), the primary literature (Barnard 1924, 1929, Hamer and Appleton 1993, 1996, Hamer et al. 1994a, b) and through direct comparison with museum material.

Dissolved oxygen, pH, electrical conductivity, turbidity, temperature and salinity were measured *in situ* on both sampling occasions using a YSI 6600–V2 multi-probe system. Waterbody dimensions (surface area and maximum depth) were estimated at each site. Maximum depth was measured at the deepest point of each waterbody using a marked depth stick. The surface area was calculated using a handheld GPS device (Garmin eTrex Vista HCx, ~ 3 m point accuracy).

An integrated 2 L surface water sample was collected from the water column at each site and thoroughly mixed, before taking a 1 L sub–sample for laboratory analysis of nutrients, suspended solids and water column chlorophyll *a*. Water samples were immediately stored in the dark below 4 °C and analysed within 24 h in the laboratory. A 300 ml subsample was taken to determine total suspended solids (TSS) and particulate organic matter (POM) in the laboratory, using APHA method 2540 as described in Eaton et al. (2005). Another 300 ml subsample from the 1 L integrated sample was filtered through 0.7 µm glass-fibre filters (Whatman GF/F). The filtrand and filtrate from each sample were stored in the dark at −19 °C until further analysis within 14 days of sampling. Soluble reactive phosphorus and ammonium were determined from the filtrate spectrophotometrically, using standard spectrophotometric methods as described by Parsons et al. (1984). Total oxidised nitrogen (the sum of nitrate and nitrite) was measured using the reduced copper cadmium method as described by Bate and Heelas (1975). Three sediment core samples (3 cm^3^ each) were collected from each site to determine sediment chlorophyll *a*. The cores were immediately placed in 90% acetone and stored in the dark at 4 °C in the field and then at -20 °C within 8 hrs of collection. Sediment and water column chlorophyll *a* and phaeopigments were extracted using 90% acetone and measured using a Turner Designs (model 10-AU) fluorometer (Welschmeyer 1994), following the standard methods of Holm–Hansen and Riemann (1978).

The presence and extent of macrophyte habitat was visually assessed qualitatively. The total cover of macrophytes (emergent and submerged) in each waterbody was recorded on an ordinal scale: 0 (not present); 1 (sparse); 2 (moderate); 3 (extensive) and 4 (complete cover). The presence and extent of floating macroalgal mats was also recorded at each site on a scale of 0–4, as for the vegetation. An estimate of the degree of agricultural land use impact within 500 m of each waterbody was visually assessed using four nominal categories: 0 (none); 1 (low); 2 (moderate); and 3 (high). The presence at each site of animals, signs of grazing, dung, and trampling was noted in order to estimate the degree of impact and place a site into one of the above categories. The sampling sites were overlain on the South African lithological map in QGIS v2.2.0 software to assess the geology underlying each site.

### Data analysis

Affinity between pairs of large branchiopod species was calculated from species co-occurrence data for both April and November samples using Fager’s index of affinity. This index (IF) indicates the likelihood that two species will co-occur in a species assemblage (Maeda-Martínez et al. 1997). The formula is as follows:

\[IF=\dfrac{2J}{n1+n2}\]

where J is the number of joint occurrences, *n*1 is the total number of occurrences of species 1 and *n*2 is the total number of occurrences of species 2. Results equal to or higher than 0.5 were considered to show affinity (Fager and McGowan 1963, see Suppl. material 1: Appendix 2 for the original data used to perform this analysis). Species data from previous collections in the region were compiled together with the current collections to provide a distribution record for the Eastern Cape Province.

In order to investigate which environmental factors best explained branchiopod assemblage composition, we related the compositional data (presence–absence) to the various environmental variables measured using distance-based Redundancy Analysis (dbRDA, Legendre and Anderson 1999, McArdle and Anderson 2001). dbRDA is a non–parametric multivariate regression procedure based on any given dissimilarity measure, in this case the Bray–Curtis coefficient (see Suppl. material 1: Appendices 3 and 4 for the original data used to perform this analysis). Environmental predictor variables were log_10_ transformed where appropriate, in order to achieve normality. For each separate sampling trip (November and April), assemblage composition as a multivariate response matrix was regressed separately either on individual environmental variables or on sets of environmental variables where appropriate (i.e. for sets of similar variables). Each environmental variable or variable set was regressed separately against assemblage composition expressed as the Bray–Curtis dissimilarity among sites. P values for dbRDA models were tested by 9999 permutations of residuals under the reduced model. Separate regressions were performed for each of the following variables or variable sets: wetland type (dam vs depression – categorical variables); underlying geology (Ecca shale, Beaufort Adelaide shale, Beaufort Tarkastad shale – categorical variables); spatial factors (latitude, longitude, altitude – continuous variables); in–wetland habitat structure (total vegetation cover, macroalgal cover – ordinal variables); surrounding land use impact (ordinal variable); dissolved oxygen, pH, temperature, turbidity (continuous variables); hydro–morphometry (depth, total surface area – continuous variables); nutrients (soluble reactive phosphorus, dissolved inorganic nitrogen – continuous variables); suspended material (total suspended solids, particulate organic matter – continuous variables); and chlorophyll *a* concentration (pelagic and benthic chlorophyll *a* – continuous variables).

Group average clustering was used to construct a dendrogram to depict the Bray-Curtis similarity of branchiopod assemblages among sites, sampling events and subregions/localities sampled. We then tested for a significant difference in species composition between the two sampling events and between subregions/localities of the Eastern Cape Karoo using nonparametric permutational MANOVA (PERMANOVA, Anderson 2001). A two–way design was employed, which incorporated the factor ‘season’ (spring – November vs autumn – April) and ‘locality’ (Aberdeen, Cradock, Jansenville, Klipplaat, Tarkastad and Mountain Zebra National Park). Residuals were permuted under a reduced model (9999 permutations). We used the zero–adjusted Bray–Curtis measure of Clarke and Gorley (2006) for calculating compositional dissimilarity among sites, given that there were some joint species absences among sites in the compositional dataset. Given the small sample size to variable ratio for each of the dbRDA, multivariate regression tests provided residual degrees of freedom ranging between 7 and 12. This possible lack of power to detect effects was countered by interpreting P values < 0.10 as offering some evidence against the null hypothesis. A standard α level of 0.05 was used for the PERMANOVA test (29 degrees of freedom). Cluster analysis and dendrogram construction were performed using PRIMER v6 software (Clarke and Warwick 2001, Clarke and Gorley 2006). dbRDA and permutational MANOVA were performed using the DISTLM and PERMANOVA routines (respectively) of the PERMANOVA+ add–on package (Anderson et al. 2008) to PRIMER v6.

## Results

### Patterns of occurrence

The species collected at each site are listed in Table 1. Large branchiopods occurred in 15 out of the 22 waterbodies investigated (i.e. 68% of the total). Thirteen species were collected in total across the two sampling events. Seven of the thirteen species were collected on both sampling events. Regarding the anostracans, four species of *Streptocephalus* were recorded, while *Branchipodopsis* was represented by a single species (Table 1). Only one notostracan, *Triops granarius* (Lucas, 1864), was recorded from the Karoo waterbodies, being present at six of the sites. Six spinicaudatan species were also collected, three of the genus *Leptestheria* and one from each of the genera *Cyzicus*, *Eocyzicus* and *Eulimnadia*. Only one laevicaudatan, *Lynceus truncatus* Barnard, 1924, was recorded. This species was found in a single small depression wetland in the Mountain Zebra National Park near Cradock (Figure 1). *Eulimnadia* sp. and *Leptestheria inermis* Barnard, 1929 were only collected in November 2014, while the other three spinicaudatans (*Eocyzicus obliquus* Sars, 1905, *Leptestheria rubidgei* Baird, 1862 and *Leptestheria striatoconcha* Barnard, 1924) and the single laevicaudatan (*Lynceus truncatus* Barnard, 1924) were only collected in April 2015.

**Table 1.** Large branchiopod species collected from 15 waterbodies of the Eastern Cape Karoo sampled in November 2014 and April 2015. See Suppl. material 1: Appendix 1 for full locality information for each site code.

Site code	A1	A2	MZ30	T2	T3	W2	W23	W25	W27	W27B	W36	W68	W93	W110	W115
Notostraca															
*Triops granarius* Lucas, 1864	+	+					+		+	+				+	+
Anostraca															
*Streptocephalus spinicaudatus* Hamer & Appleton, 1993												+			
*Streptocephalus cafer* Lovén, 1847				+	+	+		+				+			+
*Streptocephalus indistinctus* Barnard, 1924		+							+	+	+		+		+
*Streptocephalus ovamboensis* Barnard, 1924		+	+				+		+					+	
*Branchipodopsis wolfi* Daday, 1910				+				+	+						+
Laevicaudata															
*Lynceus truncatus* Barnard, 1924			+												
Spinicaudata															
*Cyzicus australis* Loven, 1847		+	+	+		+	+	+	+	+		+		+	
*Eocyzicus obliquus* Sars, 1905				+		+	+	+						+	
*Leptestheria rubidgei* Baird, 1862	+						+			+					+
*Leptestheria striatoconcha* Barnard, 1924															+
*Leptestheria inermis* Barnard, 1929											+				
*Eulimnadia* sp.									+						
**Total number of species per site**	**2**	**4**	**3**	**4**	**1**	**3**	**5**	**4**	**6**	**4**	**2**	**3**	**1**	**4**	**6**

Fourteen waterbodies (i.e. 93% of the total 15) contained at least one anostracan species (Table 1). The anostracans and spinicaudatans were the most common, occurring across 14 and 13 of the sampled waterbodies respectively. However, three of these species were represented at only a single site (*Streptocephalus spinicaudatus* Hamer & Appleton, 1993, *Leptestheria striatoconcha* and *Eulimnadia* sp.). Large branchiopod species co-occurred in 13 out of the 15 occupied sites (87%), with up to six species co-occurring within the same waterbody (sites W27 and W115, Table 1). An assemblage comprising *Triops granarius*, *Streptocephalus ovamboensis* and *Cyzicus australis* Lovén, 1847 was observed on four sites in total across both trips (Table 1) and was the most common assemblage. The Fager’s index of affinity of the different species collected is presented in Table 2. A relatively high affinity (> 0.50) with most of the species was observed for *Triops granarius*. This species co-occurred most often with *Leptestheria rubidgei* (0.73), followed by *Streptocephalus ovamboensis* (0.67), *Streptocephalus indistinctus* (0.62) and *Cyzicus australis* (0.59), while the species had the lowest affinity with *Streptocephalus cafer* (Table 2).

**Table 2.** Fager’s affinity indices between pairs of large branchiopod species in waterbodies in the Eastern Cape Karoo collected in November 2014 and April 2015.

Species	*Triops granarius*	*Streptocephalus spinicaudatus*	*Streptocephalus cafer*	*Streptocephalus indistinctus*	*Streptocephalus ovamboensis*	*Branchipodopsis wolfi*	*Lynceus truncatus*	*Cyzicus australis*	*Eocyzicus obliquus*	*Leptestheria rubidgei*	*Leptestheria striatoconcha*	*Leptestheria inermis*	*Eulimnadia* sp.
*Triops granarius*													
*Streptocephalus spinicaudatus*	0												
*Streptocephalus cafer*	0.15	0.29											
*Streptocephalus indistinctus*	**0.62**	0	0.17										
*Streptocephalus ovamboensis*	**0.67**	0	0	0.36									
*Branchipodopsis wolfi*	0.36	0	**0.6**	0.4	0.22								
*Lynceus truncatus*	0	0	0	0	0.33	0							
*Cyzicus australis*	**0.59**	0.18	**0.5**	0.38	**0.67**	0.43	0.18						
*Eocyzicus obliquus*	0.33	0	**0.55**	0	0.4	0.44	0	**0.67**					
*Leptestheria rubidgei*	**0.73**	0	0.2	0.4	0.22	0.25	0	0.29	0.22				
*Leptestheria striatoconcha*	0.25	0	0.29	0.29	0	0.4	0	0	0	0.4			
*Leptestheria inermis*	0	0	0	0.29	0	0	0	0	0	0	0		
*Eulimnadia* sp.	0.25	0	0	0.29	0.33	0.4	0	0.18	0	0	0	0	

Bold values indicate high species affinity.

### Current distribution records for the province

New and historical records for large branchiopods of the Eastern Cape are presented in Table 3. The spinicaudatan species *Cyzicus australis* is a widespread and common species in South Africa, and has previously been recorded in the Eastern Cape. During this study, the species was collected from 10 out of the 22 waterbodies (45%). The spinicaudatans *Eocyzicus obliquus* and *Leptestheria rubidgei*, collected during this study, are both known to occur in the Eastern Cape region, having previously been recorded from Hanover, Grahamstown and Port Elizabeth (Table 3). *Streptocephalus dregei* Sars, 1899, which is known to be common and widespread in the region (Hamer and Brendonck 1997), was not encountered during the current study. This was also the case with *Streptocephalus gracilis* Sars, 1898, *Streptocephalus dendyi* Barnard, 1929, *Streptocephalus cirratus* Daday, 1908, *Branchipodopsis drakensbergensis* Hamer & Appleton, 1996, *Branchipodopsis hodgsoni* Sars, 1898, *Branchipodopsis scambus* Barnard, 1929, *Eulimnadia dentatus* Barnard, 1929 and *Lynceus triangularis* Daday, 1927, which have all been previously recorded in the region.

**Table 3.** Large branchiopod distribution records for the Eastern Cape Province. Species previously recorded in the province are indicated with an asterisk. For collections made during the current study (November 2014 and April 2015), the site code is given (see Suppl. material 1: Appendix 1 for full locality information for each site code). EC = Eastern Cape.

Species	Locality & site code	Year collected	Reference
*Streptocephalus cafer* Lovén, 1847	T2; T3; W2; W25; W68; W115	2014; 2015	This study
*Streptocephalus cirratus* Daday, 1908*	Grahamstown	1998	Hamer and Martens 1998
*Streptocephalus dendyi* Barnard, 1929*	Port Elizabeth	1990	Hamer 1999
*Streptocephalus dregei* Sars, 1899*	EC	1993	Hamer 1999
*Streptocephalus gracilis* Sars, 1898*	Port Elizabeth	1898	Hamer 1999
*Streptocephalus indistinctus* Barnard, 1924	A2; W27; W27B; W36; W93;W115	2014; 2015	This study
*Streptocephalus ovamboensis* Barnard, 1924	A2; MZ30; W23; W110	2014; 2015	This study
*Streptocephalus spinicaudatus* Hamer & Appleton, 1993*	W68 Indwe/Dordrecht; Glen Avis area Dordrecht; Queenstown; Sterkstroom; Umtata Dam area	2014 1993 1998	This study Hamer and Martens 1998 Hamer 1999
*Branchipodopsis drakensbergensis* Hamer & Appleton, 1996*	Prentijiesberg	1996	Hamer 1999
*Branchipodopsis hodgsoni* Sars, 1898*	Kenton-on-Sea; Port Elizabeth	1990	Hamer 1999
*Branchipodopsis scambus* Barnard, 1929*	Grahamstown	1989	Hamer 1999
*Branchipodopsis wolfi* Daday, 1910	T2; W27; W115	2014; 2015	This study
*Cyzicus australis* Lovén, 1847*	A1; A2; MZ30; W2; W23; W25; W27; W27B; W68; W110 Port Elizabeth; Hanover; Queenstown; Molteno	2014; 2015 1847	This study Brendonck 1999
*Eocyzicus obliquus* Sars,1905*	A2; MZ30; W23; W93 Hanover	2015 1905	This study Brendonck 1999
*Eulimnadia* sp.	W27	2014	This study
*Eulimnadia dentatus* Barnard, 1929*	Hanover	1929	Brendonck 1999
*Leptestheria inermis* Barnard, 1929	W36	2014	This study
*Leptestheria rubidgei* Baird, 1862*	A1; W23; W27B; W115 Hanover; Grahamstown; Port Elizabeth	2015 1862	This study Brendonck 1999
*Leptestheria striatoconcha* Barnard, 1924	W115	2015	This study
*Lynceus triangularis* Daday, 1927 *	Port Elizabeth	1927	Brendonck 1999
*Lynceus truncatus* Barnard, 1924	MZ30	2015	This study
*Triops granarius* Lucas, 1864*	A1; A2; W23; W27; W27B; W10; W115 EC	2014; 2015	This study Rayner 1999

### Assemblage composition in relation to environmental factors

Habitat cover (macrophytes, macroalgae) was the only environmental predictor in the dbRDA regression models that was significantly related (albeit marginally) to large branchiopod assemblage composition across both seasons sampled (Table 4). Dissolved oxygen, turbidity, waterbody hydro-morphometry, suspended material and chlorophyll *a* all showed significant association with branchiopod assemblages in November 2014 only. The underlying geology of each waterbody, its position (spatial factors) and the electrical conductivity of its water were significant predictors of assemblage composition in April 2015 only. There was therefore little consistency in the environmental correlates of assemblage composition across the two seasons. The amounts of explained variation were moderate, with significant predictors explaining between 16.95% and 52.2% of the variation in branchiopod assemblage composition among sites (Table 4).

**Table 4.** Tests for relationships between the composition of large branchiopod assemblages and environmental predictor variables, either singular or in sets, using the dbRDA multivariate F-statistic. P values less than 0.10 are highlighted in bold. The column headed ‘% var’ indicates the percentage of multivariate assemblage variation (in terms of Bray-Curtis similarity) that is explained by the particular variables or sets of variables.

Variables	November 2014			April 2015		
	F	P	% var	F	P	% var
Wetland type	1.355	0.3441	13.12	1.093	0.3643	8.35
Geology	0.374	0.9074	8.56	2.014	**0.0870**	26.80
Spatial	0.767	0.6788	24.74	2.118	**0.0608**	38.85
Habitat cover	1.874	**0.0992**	31.91	1.928	**0.0956**	25.96
Land use	1.093	0.3768	10.83	0.455	0.7422	3.65
DO	4.519	**0.0016**	33.42	1.369	0.2758	10.24
pH	1.951	0.1492	17.81	0.058	0.9350	4.62
Temperature	0.512	0.7280	0.053	0.870	0.4813	6.76
Conductivity	1.584	0.2082	14.97	2.450	**0.0647**	16.95
Hydro-morphometry	1.775	0.1237	30.74	0.433	0.8488	7.29
Nutrients	1.031	0.4479	20.50	0.760	0.6299	12.14
Turbidity	4.359	**0.0042**	32.63	1.044	0.3999	8.01
Suspended material	4.369	**0.0007**	52.20	0.743	0.6692	11.91
Chlorophyll *a*	2.357	**0.0389**	37.07	0.778	0.6028	12.40

### Compositional differences between seasons and localities

**Figure 2.**
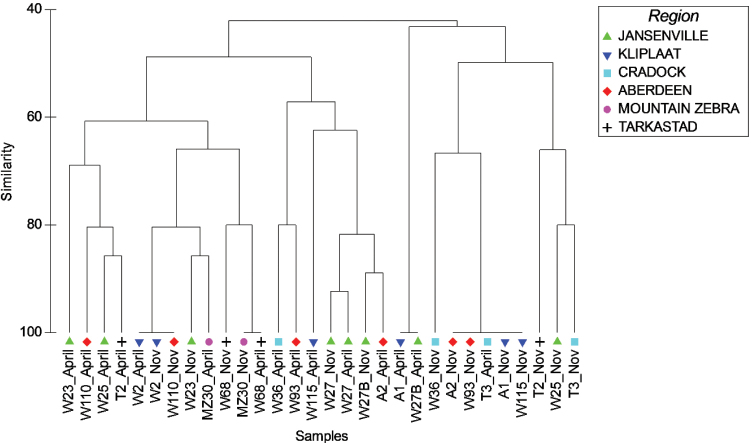
Dendrogram plot depicting the Bray-Curtis similarity of large branchiopod assemblages among sites sampled in the Eastern Cape Karoo. Sites are coded according to the season sampled (spring – November 2014 vs autumn – April 2015) and symbols indicate the sub region in which each site occurs, by reference to the nearest town name (with the exception of sites occurring within the Mountain Zebra National Park, coded as ‘Mountain Zebra’).

The dendrogram of Figure 2 depicts the similarity of sites in terms of assemblage composition between the two seasons sampled and between the various subregions of the Karoo. The sites do not appear to separate out according to either season or locality on the dendrogram, and there appears to be much assemblage variation even within each locality/subregions. The PERMANOVA test, which tests for an overall difference in multivariate space between the group centroids of each season and each locality, showed no significant overall difference between large branchiopod assemblages sampled in spring and autumn or between the localities (season: F_1,18_ = 2.262, P = 0.0807; locality: F_5,18_ = 1.707, P = 0.0632; season × locality: F_5,18_ = 0.691, P = 0.7809). The P values were low however (0.05 < P < 0.10) for both factors, suggesting some influence of these factors on assemblage composition, albeit non-significant.

## Discussion

### Large branchiopod species co-occurrence

Most study sites were inhabited by two or more species. Only two out of fifteen sites with large branchiopods contained a single *Streptocephalus* species each. The high incidence of co-occurrence is common in southern Africa. In KwaZulu-Natal, nine large branchiopod species co-occurred in a single pool (Hamer and Appleton, 1991), while eight species were collected in a small unvegetated pool in the Northern Cape (Hamer and Rayner 1996). Similar multispecies co-occurrences have been reported from Botswana (Brendonck and Riddoch 1997), the Namib Desert (Day 1990), Morocco (Thiéry 1991), India (Padhye and Dahanukar 2015), USA and Mexico (Maeda–Martínez et al. 1997, Rogers 2014a, b). Large branchiopod co-occurrence has been attributed to different life history traits, availability of biotopes and abiotic factors (Thiéry 1991, Rogers 2014a, b, 2015). In South Africa, single species occurrences have been reported from the Drakensberg Mountains and the Western Cape Province (Hamer and Martens 1998, De Roeck et al. 2007). Rogers (2014b, c, 2015) demonstrated that these vagaries of distribution are directly related to species-specific geochemical tolerance ranges. Rogers’ (2014b, c) statistical analyses demonstrated a strong correlation between pool substrate geochemistry and species or species assemblages, independent of temperature, hydroperiod, or other resources.

The only *Branchipodopsis* species found in our study, *Branchipodopsis wolfi*, always occurred together with *Streptocephalus* species. This is in contrast to the findings of Hamer and Appleton (1996), who reported that in most temporary aquatic habitats in southern Africa where *Streptocephalus* is present, *Branchipodopsis* are either absent, or are found in low numbers. There is only one known case of a multispecies *Branchipodopsis* occurrence (Barnard 1929). In that case, *Branchipodopsis drepane* Barnard, 1929, *Branchipodopsis tridens* Daday, 1910 and *Branchipodopsis wolfi* were all found in the same waterbody. The spinicaudatan *Eulimnadia* sp. was collected at a single location in our study (Table 1), co-occurring with other large branchiopod species.

### Current distribution records for the Eastern Cape Province

Previous accounts for the Eastern Cape report 14 large branchiopod species (Hamer and Brendonck 1997). Our study increases that number to 22 species, including the first record of Laevicaudata. This diversity is high compared to other areas of southern Africa. For example, only 14 species were recorded from the Western Cape (De Roeck et al. 2007) and 16 in south-eastern Zimbabwe, although in this last case a much smaller survey area was involved (Nhiwatiwa et al. 2014). Precipitation seasonality is less pronounced in the Eastern Cape than in other parts of South Africa. Most Eastern Cape areas have spring and autumn annual rainfall maxima (Stone 1988), whereas other parts of the country receive only summer or winter rainfall maxima (Mucina and Rutherford 2006). Despite this survey having been conducted during the spring and autumn annual rainfall peaks, only 13 species were collected. The nine species that were previously recorded in the province but not encountered in our study were collected mostly in the coastal areas, or outside the Karoo region. They are therefore not likely to occur in the survey area. However, the limited number of sampling trips may also have restricted our encounter probability and thus it should be emphasised that more frequent visits both intra- and inter-annually would likely reveal a more accurate picture of total diversity in the region. Furthermore, some anostracan species previously reported from the Eastern Cape may in fact have become extinct, given that they are only known from type material collected over 100 years ago (Hamer and Appleton 1996).

The anostracan *Streptocephalus cafer*, collected in 38% of the waterbodies (Table 1), is a widespread and common species in South Africa and has previously been recorded in the Eastern Cape and throughout the Karoo, while *Streptocephalus ovamboensis* is common in the arid southwest Karoo, extending north-eastwards to Groblerhoop in the Northern Cape, where rainfall is less than 300 mm per year. However, its distribution is known to vary, occurring also further east and south where rainfall is slightly higher (Hamer and Brendonck 1997; Hamer 1999). Six spinicaudatans were collected during this study, including *Eulimnadia* sp., which prior to this study had not been recorded in the Eastern Cape region. The genus is fairly widespread in South Africa, with distribution records for Heidelberg (Gauteng Province), Kimberley (Northern Cape), Greater Namaqualand (Northern Cape), Ovamboland and Kaokoveld (Namibia) (Brendonck 1999).

### New distribution records for the Eastern Cape Province

*Streptocephalus spinicaudatus* is a common species in the high altitude northern parts of the Eastern Cape (Dordrecht, Queenstown and Sterkstroom areas), where annual rainfall is higher than in the Karoo basin and peaks strongly during the summer months (Hamer 1999). Thus, our Tarkastad collection represents the westernmost distribution range for the species. Hamer and Brendonck (1997) reported that *Streptocephalus indistinctus* distribution appeared to be restricted to areas where average rainfall is > 500 mm. Thus, the species was thought to be largely excluded from the Karoo where the less than 300 mm annual rainfall is unpredictable (Hamer 1999). This species has otherwise been recorded north of 29°S in Mpumalanga and the Limpopo provinces (Hamer 1999), with only one record outside this area (Brehm 1958). Our record is the second outside its typical distribution and indicates that this species may have a wider distribution than previously believed. *Branchipodopsis wolfi* is widespread in South Africa, with distribution records from the Northern Cape, Mpumalanga and KwaZulu-Natal provinces (Hamer 1999), although prior to this study the species had not been reported from the Eastern Cape. However, this is a variable taxon that may well represent several species. Thus, there is need for molecular investigations to resolve its true status.

The *Eulimnadia* sp. record needs further analysis to determine which species occurs here. Our specimen did not have any eggs and *Eulimnadia* is only identifiable to species based on egg morphology (Rogers et al. 2012). The genus is widespread globally (Rogers et al. 2012) and in Africa (Rabet et al. 2015), but distribution data for the Eastern Cape Province prior to this study have been deficient, with records only for Heidelberg (Gauteng), Kimberley and Greater Namaqualand (Northern Cape), as well as Ovamboland and Kaokoveld (Namibia) (Brendonck 1999). *Leptestheria striatoconcha* had previously only been recorded in Heidelberg (Gauteng) and Ovamboland (Namibia). *Leptestheria inermis* had previously been recorded only between Upington and Keimoes in the Northern Cape (Brendonck 1999). Our collection extends its distribution to the Eastern Cape region, which consequently becomes the easternmost distribution range for both *Leptestheria* species. There was no Eastern Cape record of Laevicaudata prior to this study. *Lynceus truncatus* was only previously recorded in KwaZulu-Natal and Ovamboland (Barnard 1929, Rayner and Bowland 1985). We collected this species from a depressional wetland in the Mountain Zebra National Park (site MZ30).

### Environmental factors affecting assemblage composition

There was little consistency in the environmental correlates of assemblage composition across the two seasons. Habitat cover was the only variable that was significantly associated with branchiopod distribution during both sampled periods, and even these relationships were marginal (P ≈ 0.9). Thus, our data does not indicate any convincing or consistent environmental correlates of large branchiopod species composition in Eastern Cape Karoo waterbodies. This follows the prevailing sentiment of other authors, such as Rogers (2014b, 2015), who have argued that water quality data are generally of limited use in species distribution patterns, because in temporary wetlands physico-chemical parameters can fluctuate dramatically over a range of time scales. Furthermore, adaptations to highly fluctuating physico-chemical environments of arid-zone temporary wetlands may well allow some large branchiopod species to be habitat generalists, their distributions not being highly affected by local physico-chemistry. For instance, species such as *Triops granarius*, which was present at seven of our sites, is known to tolerate wide temperature and dissolved oxygen ranges. Although having some preference for warmer, muddier waters, this species appears to be a habitat generalist and can be found associated with a wide range of physico-chemical conditions (Barnard 1929, Meintjes et al. 1994).

Geology was significantly associated with species distribution during the April sampling trip. Geology underlying waterbodies affects their geochemistry and in this regard Rogers (2014b, c) found significant relationships between geochemical variables such as percent gypsum and calcium carbonate, salinity, cation type, and substrate type with anostracan species distribution across different bioregions in North America and Australia. Tuytens et al. (2015) did not find any strong effect of soil geology on the composition of anostracan and notostracan assemblages in pools in a tropical savannah habitat of south-east Zimbabwe. However, Tuytens et al. (2015) looked at a far smaller area and far fewer species than Rogers (2014b, c). During our study, large branchiopods were absent from six of the nine dams surveyed. This absence could be attributed to these systems not being sufficiently ephemeral (in fact some of the dams were semi-permanent), or perhaps they were not sampled when the active stages were present (the substrate was not sampled to determine if an egg bank was present). Large branchiopods are known for ‘bet hedging’ strategies, whereby egg banks do not necessarily hatch out on every inundation and thus could be absent from the surface water during a given sampling event, but remain in the substrate as eggs (Brendonck and De Meester 2003, Schwentner and Richter 2015, Rogers 2015a, b). Furthermore, they are known to suffer stochastic extinctions, or may not colonise successfully if the habitat is not suitable due to geochemistry, hydroperiod, natural or anthropogenic pollution, or a range of other factors (Rogers 2015).

## Conclusions

Agricultural and mining activities pose a major threat to temporary wetlands in South Africa (Hamer and Brendonck 1997, Henri et al. 2014). In our study, only one depression wetland was found in the protected Mountain Zebra National Park, whilst the other waterbodies remain at risk to potential future developments. Hydraulic fracturing is known to produce large amounts of brine wastewater, which if mismanaged may contaminate surrounding surface water. High salinities are a limiting factor for propagule hatching and survival in temporary wetlands (Cancela da Fonseca et al. 2008, Waterkeyn et al. 2008). Thus, salinisation has the potential to destroy egg banks. Additionally, climate change is a potential risk to wetlands, particularly in areas where the frequency and extent of droughts is predicted to increase (Hewitson and Crane 1996, Cubasch et al. 2013). Temporary aquatic habitats are among the most threatened globally, given that their relatively small volume and shallow depth make them extremely vulnerable to pollution, drainage and infilling (Hamer and Brendonck 1997, Williams 2006, Darwall and Brooks 2011, Collen et al. 2014). Therefore, baseline data on the distribution and ecology of keystone temporary wetland species (e.g. large branchiopods) is required in lesser studied areas, such as the Eastern Cape Karoo. It is hoped that the large branchiopod data presented in this study will contribute towards a broader biodiversity database that can be used to help inform future sustainable development in the region.

## Supplementary materials

Supplementary material 1
**Appendix 1**: Geographic position and underlying geology of the of 22 study sites. Latitude and longitude are provided in decimal degrees. The location of each site is also labelled according to the sub-region in which it occurs, by reference to the nearest town name. An exception to this is MZNP, which represents sites sampled within the Mountain Zebra National Park and sites sampled along the R337 road, which are located in mountainous terrain with no nearby towns.
**Appendix 2**: Large branchiopod species found in November 2014 and April 2015 in the 15 study sites included in the survey. a = present only in April; n = present only in November; an = present during both periods; 0 = absent during both periods.
**Appendix 3**: Environmental characteristics of the 22 waterbodies investigated in November 2014. All physico-chemical and biological values are means taken from 3 subsamples for each site. D – dam; ND – Natural depression; R – river; CV – complex vegetation; SV – simple vegetation; BU – benthic unvegetated; LU – land-use impact; DO – dissolved oxygen; Temp. – temperature; Cond. – conductivity; DIP – dissolved inorganic phosphorus; DIN – dissolved inorganic nitrogen; TSS – total suspended solids; POM – particulate organic matter; P chl-a – pelagic chlorophyll a; B chl-a – benthic chlorophyll a; MD – maximum depth; SA – surface area.
**Appendix 4**: Environmental characteristics of the 22 waterbodies investigated in April 2015. All physico-chemical and biological values are means taken from 3 subsamples for each site. D – dam; ND – Natural depression; R – river; CV – complex vegetation; SV – simple vegetation; BU – benthic unvegetated; LU – land-use impact; DO – dissolved oxygen; Temp. – temperature; Cond. – conductivity; DIP – dissolved inorganic phosphorus; DIN – dissolved inorganic nitrogen; TSS – total suspended solids; POM – particulate organic matter; P chl-a – pelagic chlorophyll a; B chl-a – benthic chlorophyll a; MD – maximum depth; SA – surface area. This dataset is made available under the Open Database License (http://opendatacommons.org/licenses/odbl/1.0/). The Open Database License (ODbL) is a license agreement intended to allow users to freely share, modify, and use this Dataset while maintaining this same freedom for others, provided that the original source and author(s) are credited.Annah Mabidi, Matthew S. Bird, Renzo Perissinotto, D. Christopher Rogers
