# Supplementary material for: Ecology and distribution of large branchiopods (Crustacea, Branchiopoda, Anostraca, Notostraca, Laevicaudata, Spinicaudata) of the Eastern Cape Karoo, South Africa
Source: Zookeys. 2016 Sep 19;(618):15–38. doi: 10.3897/zookeys.618.9212 (PMC5102048; doi:10.3897/zookeys.618.9212)
Supplement: Supplementary material 1 [file zookeys-618-015-s001.docx]

**Supplementary material**

**Appendix 1.** Geographic position and underlying geology of the of 22 study sites. Latitude and longitude are provided in decimal degrees. The location of each site is also labelled according to the sub-region in which it occurs, by reference to the nearest town name. An exception to this is MZNP, which represents sites sampled within the Mountain Zebra National Park and sites sampled along the R337 road, which are located in mountainous terrain with no nearby towns.

| Water body type | Site code | Location | Latitude | Longitude | Altitude (masl) | Underlying geology |
| --- | --- | --- | --- | --- | --- | --- |
|  |  |  |  |  |  |  |
| Dam | A1 | Klipplaat | -32.921278 | 24.260071 | 674 | Ecca |
| Dam | W23 | Jansenville | -32.626008 | 24.684467 | 582 | Beaufort Adelaide |
| Dam | W36 | Cradock | -32.003417 | 25.142233 | 1194 | Beaufort Adelaide |
| Dam | W6 | Jansenville | -32.999809 | 24.505765 | 537 | Ecca |
| Dam | MZ27 | MZNP | -32.237188 | 25.460087 | 1228 | Beaufort Adelaide |
| Dam | W56 | R337 road | -32.491048 | 25.318032 | 1175 | Beaufort Adelaide |
| Dam | W87 | Camdeboo | -32.294236 | 24.274062 | 796 | Beaufort Adelaide |
| Dam | W45 | R337 road | -32.316885 | 24.679974 | 1311 | Beaufort Adelaide |
| Dam | T1 | Tarkastad | -31.949062 | 26.18231 | 1279 | Beaufort Tarkastad |
| Depression wetland | A2 | Aberdeen | -32.619112 | 24.183249 | 731 | Beaufort Adelaide |
| Depression wetland | MZ30 | MZNP | -32.149391 | 25.49982 | 1008 | Beaufort Adelaide |
| Depression wetland | W25 | Jansenville | -32.603381 | 24.651856 | 598 | Beaufort Adelaide |
| Depression wetland | T2 | Tarkastad | -31.932568 | 26.079092 | 1217 | Beaufort Tarkastad |
| Depression wetland | T3 | Tarkastad | -32.166526 | 25.676791 | 930 | Beaufort Tarkastad |
| Depression wetland | W2 | Klipplaat | -33.027906 | 25.494876 | 616 | Ecca |
| Depression wetland | W27 | Jansenville | -32.494572 | 24.299996 | 661 | Beaufort Adelaide |
| Depression wetland | W27B | Jansenville | -32.507438 | 24.655214 | 654 | Beaufort Adelaide |
| Depression wetland | W68 | Tarkastad | -31.955212 | 26.196083 | 1275 | Beaufort Adelaide |
| Depression wetland | W93 | Aberdeen | -32.457843 | 24.094083 | 738 | Ecca |
| Depression wetland | W110 | Aberdeen | -32.626712 | 24.192674 | 725 | Beaufort Adelaide |
| Depression wetland | W115 | Klipplaat | -32.943724 | 24.265472 | 669 | Beaufort Adelaide |
| Depression wetland | W117 | Klipplaat | -32.981841 | 24.345178 | 641 | Ecca |
|  |  |  |  |  |  |  |

**Appendix 2.** Large branchiopod species found in November 2014 and April 2015 in the 15 study sites included in the survey. a = present only in April; n = present only in November; an = present during both periods; 0 = absent during both periods.

| **Species / Site code** | A1 | A2 | MZ30 | T2 | T3 | W2 | W23 | W25 | W27 | W27B | W36 | W68 | W93 | W110 | W115 |
| --- | --- | --- | --- | --- | --- | --- | --- | --- | --- | --- | --- | --- | --- | --- | --- |
| *Triops granarius* | a | a | 0 | 0 | 0 | 0 | a | 0 | an | an | 0 | 0 | 0 | a | a |
| *Streptocephalus spinicaudatus* | 0 | 0 | 0 | 0 | 0 | 0 | 0 | 0 | 0 | 0 | 0 | n | 0 | 0 | 0 |
| *Streptocephalus cafer* | 0 | 0 | 0 | n | n | a | 0 | an | 0 | 0 | a | 0 | 0 | 0 | a |
| *Streptocephalus indistinctus* | 0 | a | 0 | 0 | 0 | 0 | 0 | 0 | n | n | a | 0 | a | 0 | a |
| *Streptocephalus ovamboensis* | 0 | a | a | 0 | 0 | 0 | an | 0 | an | 0 | 0 | 0 | a | n | 0 |
| *Branchipodopsis wolfi* | 0 | 0 | 0 | n | 0 | 0 | 0 | n | n | 0 | 0 | 0 | 0 | 0 | a |
| *Lynceus truncatus* | 0 | 0 | a | 0 | 0 | 0 | 0 | 0 | 0 | 0 | 0 | 0 | 0 | 0 | 0 |
| *Cyzicus australis* | 0 | a | an | an | 0 | an | an | a | n | n | 0 | an | 0 | an | 0 |
| *Eocyzicus obliquus* | 0 | 0 | 0 | a | 0 | a | a | a | 0 | 0 | 0 | 0 | 0 | a | 0 |
| *Leptestheria rubidgei* | a | 0 | 0 | 0 | 0 | 0 | a | 0 | 0 | a | 0 | 0 | 0 | 0 | a |
| *Leptestheria striatoconcha* | 0 | 0 | 0 | 0 | 0 | 0 | 0 | 0 | 0 | 0 | 0 | 0 | 0 | 0 | a |
| *Leptestheria inermis* | 0 | 0 | 0 | 0 | 0 | 0 | 0 | 0 | 0 | 0 | n | 0 | 0 | 0 | 0 |
| *Eulimnadia* sp. | 0 | 0 | 0 | 0 | 0 | 0 | 0 | 0 | n | 0 | 0 | 0 | 0 | 0 | 0 |

**Appendix 3.** Environmental characteristics of the 22 waterbodies investigated in November 2014. All physico-chemical and biological values are means taken from 3 subsamples for each site. D – dam; ND – Natural depression; R – river; CV – complex vegetation; SV – simple vegetation; BU – benthic unvegetated; LU – land-use impact; DO – dissolved oxygen; Temp. – temperature; Cond. – conductivity; DIP– dissolved inorganic phosphorus; DIN – dissolved inorganic nitrogen; TSS – total suspended solids; POM – particulate organic matter; P chl-*a* – pelagic chlorophyll *a*; B chl-*a* – benthic chlorophyll *a*; MD – maximum depth; SA – surface area.

**(Continued overleaf)**

| Site code | Waterbody type | CV | SV | BU | MA | LU | DO  (mg L^−1^) | pH | Temp.  (^o^C) | Cond.  (mS cm^−1^) | DIP (µM) | DIN  (µM) | Turbidity (NTU) |  | TSS  (mg L^−1^) | POM (%) | P chl-*a* (mg m^-3^) | B chl-*a*  (mg m^-2^) | MD (m) | SA (m^2^) |
| --- | --- | --- | --- | --- | --- | --- | --- | --- | --- | --- | --- | --- | --- | --- | --- | --- | --- | --- | --- | --- |
| A1 | D | 0 | 0 | 4 | 0 | 2 | 6.60 | 7.68 | 22.81 | 0.05 | 9.62 | 2.86 | 155.10 |  | 50.10 | 17 | 0.11 | 6.51 | 0.80 | 3384.4 |
| MZ27 | D | 1 | 0 | 3 | 0 | 1 | 7.48 | 8.90 | 22.68 | 0.31 | 0.06 | 1.43 | 10.20 |  | 11.87 | 61 | 0.64 | 13.78 | 3.53 | 67809 |
| T1 | D | 2 | 0 | 2 | 0 | 2 | 8.71 | 8.39 | 26.81 | 0.10 | 2.00 | 1.43 | 75.80 |  | 56.80 | 30 | 48.48 | 42.46 | 0.52 | 1852.7 |
| W6 | D | 0 | 2 | 2 | 0 | 3 | 20.41 | 10.03 | 26.06 | 0.16 | 8.07 | 5.00 | 292.80 |  | 190.60 | 65 | 68.64 | 58.35 | 0.80 | 4163.8 |
| W23 | D | 0 | 0 | 4 | 0 | 3 | 7.13 | 8.22 | 26.89 | 0.34 | 14.63 | 182.77 | 745.30 |  | 47.08 | 20 | 6.20 | 36.92 | 0.35 | 5366.3 |
| W36 | D | 3 | 0 | 1 | 3 | 2 | 8.97 | 8.40 | 26.06 | 0.48 | 0.71 | 1.43 | 1.10 |  | 3.11 | 70 | 2.89 | 1006.81 | 1.47 | 16713.5 |
| W45 | D | 4 | 0 | 0 | 3 | 2 | 14.53 | 10.17 | 24.03 | 0.32 | 0.10 | 0.71 | 2.20 |  | 3.48 | 87 | 1.36 | 281.70 | 3.17 | 33859.2 |
| W56 | D | 3 | 0 | 1 | 3 | 2 | 13.33 | 10.52 | 22.42 | 0.22 | 0.77 | 8.57 | 15.00 |  | 19.03 | 32 | 15.63 | 57.42 | 1.57 | 18738.7 |
| W87 | D | 4 | 0 | 0 | 3 | 2 | 9.30 | 10.64 | 22.80 | 0.87 | 0.23 | 3.57 | 0.00 |  | 4.08 | 65 | 1.53 | 69.33 | 1.41 | 22557.0 |
| A2 | ND | 2 | 0 | 2 | 0 | 1 | 9.96 | 9.08 | 23.09 | 0.04 | 2.68 | 2.86 | 132.80 |  | 68.23 | 37 | 104.96 | 22.44 | 0.58 | 3384.1 |
| MZ30 | ND | 0 | 0 | 4 | 0 | 0 | 6.07 | 8.53 | 27.74 | 0.10 | 3.29 | 72.82 | 1282.70 |  | 496.50 | 11 | 71.04 | 10.17 | 0.13 | 1505.8 |
| T2 | ND | 3 | 0 | 1 | 2 | 2 | 8.11 | 7.87 | 30.93 | 0.11 | 5.75 | 2.14 | 76.30 |  | 17.68 | 52 | 24.90 | 57.74 | 0.38 | 326.2 |
| T3 | ND | 0 | 0 | 4 | 0 | 3 | 14.81 | 9.90 | 28.70 | 0.05 | 1.58 | 10.00 | 49.10 |  | 41.30 | 79 | 95.68 | 33.36 | 0.50 | 2100.0 |
| W2 | ND | 2 | 0 | 2 | 0 | 3 | 9.28 | 8.48 | 22.03 | 0.15 | 2.68 | 6.43 | 168.70 |  | 60.67 | 20 | 5.06 | 19.42 | 0.41 | 668.8 |
| W25 | ND | 4 | 0 | 0 | 3 | 2 | 9.09 | 8.68 | 27.77 | 0.08 | 0.81 | 0.71 | 14.00 |  | 4.63 | 92 | 6.44 | 95.56 | 0.40 | 192.0 |
| W27 | ND | 1 | 0 | 3 | 0 | 2 | 5.27 | 8.28 | 18.19 | 0.19 | 4.39 | 5.00 | 62.10 |  | 56.68 | 16 | 13.68 | 41.32 | 0.29 | 1343.7 |
| W27B | ND | 1 | 0 | 3 | 0 | 2 | 4.16 | 8.53 | 29.29 | 0.08 | 12.72 | 5.00 | 152.80 |  | 68.23 | 13 | 4.82 | 8.93 | 1.10 | 3053.5 |
| W68 | ND | 0 | 2 | 2 | 0 | 2 | 8.06 | 8.52 | 23.79 | 0.04 | 1.90 | 6.43 | 229.60 |  | 38.60 | 21 | 27.62 | 12.71 | 0.60 | 2096.9 |
| W93 | ND | 1 | 0 | 3 | 0 | 2 | 11.43 | 9.61 | 29.73 | 0.17 | 1.68 | 7.14 | 1315.00 |  | 108.44 | 11 | 0.30 | 36.03 | 0.10 | 401.2 |
| W110 | ND | 0 | 0 | 4 | 0 | 3 | 7.80 | 8.74 | 23.23 | 0.22 | 18.69 | 25.70 | 1257.40 |  | 164.77 | 10 | 30.37 | 33.07 | 0.25 | 6158.0 |
| W115 | ND | 3 | 0 | 1 | 0 | 2 | 6.32 | 7.56 | 26.43 | 0.09 | 13.82 | 0.71 | 38.00 |  | 11.55 | 29 | 4.42 | 11.73 | 0.56 | 1136.5 |
| W117 | ND | 1 | 0 | 3 | 0 | 1 | 8.13 | 8.51 | 23.48 | 0.05 | 14.66 | 2.14 | 92.50 |  | 25.35 | 19 | 0.19 | 4.35 | 0.45 | 580.9 |
|  |  |  |  |  |  |  |  |  |  |  |  |  |  |  |  |  |  |  |  |  |
|  |  |  |  |  |  |  |  |  |  |  |  |  |  |  |  |  |  |  |  |  |

**Appendix 3 (continued)**

**Appendix 4.** Environmental characteristics of the 22 waterbodies investigated in April 2015. All physico-chemical and biological values are means taken from 3 subsamples for each site. D – dam; ND – Natural depression; R – river; CV – complex vegetation; SV – simple vegetation; BU – benthic unvegetated; LU – land-use impact; DO – dissolved oxygen; Temp. – temperature; Cond. – conductivity; DIP– dissolved inorganic phosphorus; DIN – dissolved inorganic nitrogen; TSS – total suspended solids; POM – particulate organic matter; P chl-*a* – pelagic chlorophyll *a*; B chl-*a* – benthic chlorophyll *a*; MD – maximum depth; SA – surface area.

**(Continued overleaf)**

| Site code | Water body type | CV | SV | BU | MA | LU | DO  (mg L^−1^) | pH | Temp  (^o^C) | Cond.  (mS cm^−1^) | DIP (µM) | DIN  (µM) | Turbidity (NTU) | TSS  (mg L^−1^) | POM (%) | P chl-*a* (mg m^-3^) | B chl-*a*  (mg m^-2^) | MD  (m) | SA (m^2^) |
| --- | --- | --- | --- | --- | --- | --- | --- | --- | --- | --- | --- | --- | --- | --- | --- | --- | --- | --- | --- |
| A1 | D | 0 | 0 | 4 | 0 | 2 | 4.94 | 8.33 | 24.64 | 0.08 | 8.36 | 71.39 | 478.00 | 36.22 | 12 | 4.46 | 7.35 | 0.30 | 2716.80 |
| MZ27 | D | 1 | 0 | 3 | 0 | 1 | 5.77 | 8.50 | 18.11 | 0.32 | 4.94 | 12.14 | 14.00 | 5.80 | 72 | 1.74 | 29.95 | 3.55 | 69052.00 |
| T1 | D | 2 | 0 | 2 | 0 | 2 | 5.22 | 7.66 | 20.52 | 0.06 | 2.16 | 17.85 | 440.00 | 131.82 | 31 | 13.12 | 32.44 | 0.68 | 1190.30 |
| W6 | D | 0 | 2 | 2 | 0 | 3 | 7.59 | 9.14 | 26.36 | 0.20 | 6.59 | 19.28 | 91.20 | 32.75 | 31 | 36.04 | 44.15 | 1.00 | 2980.00 |
| W23 | D | 0 | 0 | 4 | 0 | 3 | 5.27 | 8.63 | 21.74 | 0.12 | 10.49 | 2.14 | 192.00 | 20.02 | 17 | 2.32 | 57.30 | 1.20 | 16485.00 |
| W36 | D | 3 | 0 | 1 | 0 | 2 | 4.64 | 8.39 | 19.79 | 0.39 | 2.40 | 0.00 | 13.20 | 6.37 | 60 | 8.99 | 353.01 | 1.37 | 11684.00 |
| W45 | D | 4 | 0 | 0 | 3 | 2 | 3.32 | 9.22 | 17.86 | 0.29 | 3.68 | 7.85 | 21.80 | 2.82 | 40 | 5.99 | 83.08 | 3.53 | 35111.00 |
| W56 | D | 1 | 0 | 3 | 3 | 2 | 11.97 | 10.12 | 23.48 | 0.27 | 32.38 | 33.56 | 21.40 | 39.73 | 20 | 32.16 | 221.54 | 1.33 | 11072.00 |
| W87 | D | 0 | 0 | 4 | 1 | 2 | 1.48 | 8.58 | 18.02 | 1.59 | 13.40 | 162.78 | 452.00 | 98.83 | 21 | 6.38 | 223.77 | 0.53 | 9939.40 |
| A2 | ND | 2 | 0 | 2 | 0 | 1 | 1.75 | 7.20 | 19.29 | 0.08 | 9.62 | 52.12 | 781.00 | 65.53 | 9 | 4.28 | 16.46 | 0.36 | 1448.50 |
| MZ30 | ND | 0 | 0 | 4 | 0 | 0 | 3.85 | 7.61 | 17.42 | 0.07 | 3.52 | 2.14 | 1000.00 | 1255.39 | 5 | 24.06 | 24.41 | 0.60 | 3957.40 |
| T2 | ND | 3 | 0 | 1 | 3 | 2 | 7.03 | 7.43 | 22.80 | 0.06 | 0.16 | 0.00 | 140.00 | 102.58 | 18 | 11.24 | 31.13 | 0.35 | 347.40 |
| T3 | ND | 0 | 0 | 4 | 0 | 3 | 5.31 | 7.84 | 24.04 | 0.19 | 0.00 | 0.00 | 1000.00 | 520.15 | 17 | 20.52 | 69.68 | 0.28 | 1260.90 |
| W2 | ND | 2 | 0 | 2 | 0 | 3 | 2.34 | 7.34 | 23.92 | 0.16 | 1.58 | 0.71 | 832.00 | 126.25 | 8 | 4.98 | 18.02 | 0.54 | 995.67 |
| W25 | ND | 4 | 0 | 0 | 2 | 2 | 4.99 | 8.45 | 25.83 | 0.07 | 14.40 | 9.28 | 8.20 | 4.55 | 94 | 14.32 | 29.35 | 0.33 | 173.16 |
| W27 | ND | 1 | 0 | 3 | 1 | 2 | 3.86 | 7.90 | 22.48 | 0.19 | 2.03 | 0.00 | 620.00 | 92.20 | 11 | 7.25 | 37.88 | 0.20 | 1442.90 |
| W27B | ND | 1 | 0 | 3 | 0 | 2 | 4.02 | 7.46 | 19.95 | 0.08 | 3.75 | 0.00 | 87.70 | 3.35 | 73 | 7.-2 | 110.89 | 1.30 | 2809.60 |
| W68 | ND | 0 | 2 | 2 | 0 | 2 | 3.77 | 8.51 | 18.66 | 0.04 | 1.84 | 5.72 | 68.00 | 14.07 | 40 | 6.72 | 16.74 | 1.23 | 2100.30 |
| W93 | ND | 1 | 0 | 3 | 0 | 2 | 4.25 | 8.06 | 19.77 | 0.15 | 0.45 | 0.71 | 138.00 | 19.03 | 22 | 7.19 | 48.70 | 0.78 | 1053.00 |
| W110 | ND | 0 | 0 | 4 | 0 | 3 | 4.49 | 7.65 | 18.66 | 0.16 | 12.37 | 155.64 | 944.00 | 52.87 | 9 | 5.00 | 299.85 | 0.61 | 8472.10 |
| W115 | ND | 3 | 0 | 1 | 0 | 2 | 1.18 | 7.27 | 23.24 | 0.11 | 0.00 | 0.00 | 388.00 | 50.22 | 12 | 7.64 | 8.01 | 0.60 | 1043.50 |

**Appendix 4 (continued)**
